# A Video Interview With William B. Adams, Jr., MD

**DOI:** 10.1093/asjof/ojab053

**Published:** 2021-12-13

**Authors:** Nicole R Vingan, William P Adams, Jeffrey M Kenkel

**Affiliations:** Department of Plastic Surgery, University of Texas Southwestern Medical Center, Dallas, TX, USA

*Second Thoughts on First Thoughts* is a new edition to *Aesthetic Surgery Journal Open Forum* aimed at exploring the evolution of a surgeon’s practice over time. This interview-based installment is intended to elucidate wisdom gleaned from experienced plastic surgeons who have reflected upon and modified certain aspects of their craft. What can be aptly labeled as the “surgeon’s learning curve," these improvements can provide perspective and insights among colleagues who have tweaked their strategies over time to achieve better results.^1,2^ In this article, we will explore the evolution of William B. Adams’, MD, workflow in his surgical practice and how he has developed strategies to optimize time with his patients. 

## WHAT I USED TO DO

Dr Adams noted that at the start of practice, for many years, he would typically operate and see clinic patients on the same day (Video). Additionally, he would see both “new” and “follow-up” patients while in the clinic.

## WHY I MADE A CHANGE AND HOW I DO IT NOW

Dr Adams’ second thoughts were prompted through experience and monitoring of workflow in practice. He observed how, despite his best abilities, the combination of surgical and clinic days, as well as varied patient status throughout those days, could lead him to fall behind and have patients waiting longer than he desired. This impelled him to restructure his schedule to utilize time more efficiently. Dr Adams restructured in such a way that he now schedules surgical and clinic days separately. He also schedules clinic patients based on status as “new” or “follow-up” patients. Using this revised system, he now schedules “new” patients on the same day once per week, “follow-up” patients on the same day once per week, along with small, in-office procedures, and then operates the remainder of days. The updated schedule allows for consistency and a better workflow from the start until the end of the day. The mindset that he and his staff are able to maintain throughout each designated day allows the clinic to more reliably run on schedule, thereby ensuring that his patients do not have long wait times. A counterargument to this practice change could be that patients have concerns about a limiting clinic schedule; however, despite initial apprehension from Dr Adams on this very notion, this has not been a common issue. When applicable, alternative appointments can be made to accommodate those who still wish to be seen.

## SUMMARY

The quintessence of our success as surgeons is the patient experience. Despite our best efforts to multitask, it is almost impossible to be in all the places we are needed at once. For Dr Adams, the comfort, joy, and ease he can provide his patients are of utmost importance. Seeing patients efficiently and effectively and also keeping them happy are the lifeline of his practice. Rather than feel burdened or limited by his former setup, Dr Adams chose to reconsider his workflow; in doing so, he has created an efficient work schema resulting in a well-organized work and patient care environment.
